# Clinical outcomes and safety of polymyxin B in the treatment of carbapenem-resistant Gram-negative bacterial infections: a real-world multicenter study

**DOI:** 10.1186/s12967-021-03111-x

**Published:** 2021-10-16

**Authors:** Xiaojuan Zhang, Shaoyan Qi, Xiaoguang Duan, Bing Han, Shuguang Zhang, Shaohua Liu, Haixu Wang, Haibo Zhang, Tongwen Sun

**Affiliations:** 1 Department of General ICU, The First Affiliated Hospital of Zhengzhou University, Henan Key Laboratory of Critical Care Medicine, Zhengzhou Key Laboratory of Sepsis, Henan Engineering Research Center for Critical Care Medicine, Zhengzhou, Henan China; 2 Department of ICU, The Second Affiliated Hospital of Zhengzhou University, Zhengzhou, Henan China; 3 Interdepartmental Division of Critical Care Medicine, Departments of Anesthesia and Physiology, University of Toronto, Room 619, LKSKI, 30 Bond Street, Toronto, ON M5B 1W8 Canada

**Keywords:** Carbapenem-resistant Gram-negative bacilli, Infections, Polymyxin B, Adverse effects, 28-day mortality

## Abstract

**Background:**

High morbidity and mortality due to carbapenem-resistant Gram-negative bacilli (CR-GNB) has led to the resurgence of polymyxin B (PMB) use in the last decade. The aim of our multicenter, real-world study was to evaluate the effectiveness and safety of PMB in the treatment of CR-GNB infections.

**Methods:**

The real-world study included patients treated with intravenous PMB for at least 7 days during the period of October 2018 through June 2019. Associations between these clinical features and 28-day mortality or all-cause hospital mortality were explored through univariate analyses and multivariable logistic regression.

**Results:**

The study included 100 patients. Many patients presented with combined chronic conditions, septic shock, mechanical ventilation, and the presence of *Klebsiella pneumoniae*. The mean duration of PMB therapy was 11 days (range 7–38 days). Temperature (38 °C vs 37.1 °C), white blood cells (14.13 × 10^9^/l vs 9.28 × 10^9^/l), C-reactive protein (103.55 ug/l vs 47.60 ug/l), procalcitonin (3.89 ng/ml vs 1.70 ng/ml) and APACHE II levels (17.75 ± 7.69 vs 15.98 ± 7.95) were significantly decreased after PMB treatment. The bacteria eradication rate was 77.65%. The overall mortality at discharge was 15%, and 28-day mortality was 40%. Major adverse reactions occurred in 16 patients. Nephrotoxicity was observed in 7 patients (7%).

**Conclusions:**

Our results provide positive clinical and safety outcomes for PMB in the treatment of CR-GNB. Timely and appropriate use of PMB may be particularly useful in treating patients with sepsis in CR-GNB infections.

## Introduction

In recent years, infections due to carbapenem-resistant Gram-negative bacilli (CR-GNB) have become an increasingly important cause of mortality and morbidity around the world [1]. The organisms most commonly identified in CR-GNB infections are *Klebsiella pneumoniae*, *Acinetobacter baumannii*, and *Pseudomonas aeruginosa* [2–4]. These bacteria can lead to bloodstream, respiratory tract, skin and soft tissue, urinary tract, intra-abdominal, and surgical infections [3, 5–7]. They are responsible for nosocomial infections, particularly among critically ill patients hospitalized in intensive care units (ICUs) [8].

Mortality rates of greater than 47% have been reported for CR-GNB infections [5, 9–13]. The decline in the development of newer antibiotics has created a challenge for clinicians treating CR-GNB infections [2, 14]. As a result, physicians have sought solutions in the arsenal of older therapeutics. This has led to the re-introduction of polymyxins in the treatment of infections caused by CR-GNB, as polymyxins are one of the few antibiotics that remain effective against these organisms [3]. Two polymyxins in clinical use, polymyxin B (PMB) and colistin, had fallen out of favor due to nephrotoxicity and neurotoxicity reported during 1960s. However, due to multiple drug resistance among Gram-negative bacilli, physicians have been increasingly forced to rely on polymyxins for the treatment of infections caused by these pathogens. It has not been determined which of these agents is superior in terms of the cure rate or microbiological resolution [15, 16]. A systematic review and meta-analysis summarized findings that included no significant difference in mortality between patients treated with these two polymyxins; this study also found a lower nephrotoxicity profile for PMB [15]. Additionally, recently published research demonstrated that PMB, unlike colistin, is not cleared renally and therefore, dosing of PMB should not be adjusted based on renal function [17]. However, the simulated values for AUC across 24 h at steady state for patients with creatinine clearance ≥ 80 ml/min were higher than those for patients with creatinine clearance < 80 ml/min [18].

The international consensus guidelines for the optimal use of polymyxins recommend that patients intravenous PMB should receive a dose of 1.25–1.5 mg/kg (equivalent to 12,500–15,000 IU/kg) PMB every 12 h infused over 1 h [16]. In one study, clinicians found that combination therapy with at least two in vitro active agents was associated with higher efficacy in treating bloodstream infections caused by CR-GNB [6, 19]. Carbapenem was the agent combined most commonly with PMB, followed by tigecycline, and cefoperazone–sulbactam [20].

Currently, there is a lack of data available on the efficacy, 28-day mortality, and adverse events for PMB in the treatment of CR-GNB infections. Here, we report on a multicenter, real-world study of patients receiving intravenous PMB to investigate the clinical outcomes of antimicrobial therapy in patients infected with CR-GNB.

## Methods

### Study design and patients

This multicenter, real-world study was conducted at 14 hospitals in Henan province during the period of October 2018 through June 2019. The institutional research ethics committee of the First Affiliated Hospital of Zhengzhou University approved the retrospective study (SS-2019-015).

#### Inclusion criteria

Patients aged > 14 years with CR-GNB infection who received PMB therapy for at least 7 days were included in the study. Patients with positive culture of CR-GNB, or patients were highly suspected infection of CR-GNB would receive intravenous PMB. The organisms identified in CR-GNB infections are *Klebsiella pneumoniae*, *Acinetobacter baumannii*, *Pseudomonas aeruginosa*, *Escherichia coli,* and *Stenotrophomonas maltophilia.*

Exclusion criteria were < 14 years old, received intravenous PMB for fewer than 7 days, previous enrolment in the trial, pregnancy, breast feeding during the study period, or on renal replacement therapy.

### Data collection

Data was collected from electronic patient registration and follow-up. The database was generated by the clinician through a query of the electronic medical records. The following variables were recorded: age, gender, underlying disease, hospitalization date, dates of admission to and discharge from ICU, vital signs, Acute Physiology and Chronic Health Evaluation II (APACHEII) score, Sequential Organ Failure Assessment (SOFA) score, any major surgeries performed, ventilator care, site of isolation of organisms, exposure to antimicrobial therapies, clinical features, biochemical indices, and microbiological data on admission and on the day of introduction of PMB.

The dose and duration of PMB therapy, renal function, clinical and microbiological outcomes, and adverse reactions to PMB were noted. Patients were followed up until the end of treatment at 28 days.

### Patient screening procedure

Diagnoses of infections were based on clinical features and the isolation of bacteria from areas that are normally sterile. The microbiologically documented infection was defined as positive cultures in sterile of localized, and absence of any bacterial pollution or colonization. Severe sepsis was defined as sepsis associated with organ dysfunction or hypoperfusion. Septic shock was defined as sepsis 3.0 [21]. Pulmonary infection included hospital-associated pneumonia (HAP) and ventilator-associated pneumonia (VAP). HAP was defined as a pneumonia occurring 48 h or more after admission. VAP was defined as a pneumonia developing 48 h or more after tracheal intubation. Chronic diseases included heart disease, hypertension, stroke, cancer, diabetes mellitus, and chronic obstructive pulmonary disease.

### Microbiology

The CR-GNB include *Enterobacteriaceae, Acinetobacter* spp.,* Pseudomonas aeruginosa, Stenotrophomonas maltophilia,* and *Burkholderia cepacia.* But, the most common CR-GNB found were *Klebsiella pneumoniae*, *Acinetobacter baumannii*, *Pseudomonas aeruginosa*, *Escherichia coli*, and *Stenotrophomonas maltophilia.* All CR-GNB infections were identified in the microbiology laboratory. The biological samples included blood, vein catheter samples, urine, sputum, tracheal secretions, bronchial‐alveolar lavage fluid, intraperitoneal fluid, and pleural drainage fluid. Bacterial identification and drug sensitivity tests were performed using a Vitek^®^ 2 automated system (France Biomerieux). Susceptibility was interpreted according to Clinical and Laboratory Standards Institute criteria [22]. *Enterobacteriaceae* with a minimal inhibitory concentration (MIC) ≥ 4 µg/ml were considered resistance to carbapenem [22]. *Pseudomonas aeruginosa* and *Acinetobacter* spp. with a minimal inhibitory concentration (MIC) ≥ 8 µg/ml were considered resistance to carbapenem [22]. *Burkholderia cepacia* with a MIC ≥ 16 µg/ml were considered resistance to meropenem [22]. Isolates with a MIC ≤ 2 µg/ml were considered susceptible to PMB (colistin breakpoint for *Enterobacteriaceae*) [23]. The treating clinicians evaluated whether pathogens were the pathogenic bacteria according to the characteristics of pathogen distribution in the institution and their own experience.

### Treatment regimen

All patients were treated with intravenous PMB, most in combination with other anti-CR-GNB agents, to which all strains remained sensitive. The international consensus guidelines for the optimal use of polymyxins recommend that patients who require intravenous PMB receive a loading dose of 2.0–2.5 mg/kg, and then a dose of 1.25–1.5 mg/kg (equivalent to 12,500–15,000 IU/kg) PMB every 12 h infused over 1 h [16]. Upon isolation of strains of CR-GNB that were resistant to carbapenem, an intravenous antibiotic regimen was initiated at the discretion of the attending physician.

### Outcomes

The primary outcome of this analysis was 28-day mortality; the secondary outcomes included all-cause hospital mortality, ICU mortality, and the occurrence of adverse events during PMB therapy. The clinical outcomes of this study were based on the recovery of patients following PMB therapy. The measure of 28‐day mortality refers to patient deaths occurring within 28 days from the start of intravenous PMB, even if the death was related to other comorbidities that were not the infection.

Clinical cure was defined as a combined outcome of survival and the complete disappearance or improvement of signs and symptoms of infection after day 7 of PMB therapy. Failure of treatment was defined as maintenance or worsening of signs and symptoms of disease or radiologic deterioration. Bacteria eradication rate, the rate that the bacteria were eliminated during the course of PMB treatment.

Common adverse events included nephrotoxicity, neurotoxicity, skin hyperpigmentation, and eosinophil increase. The major adverse reaction was nephrotoxicity. Nephrotoxicity was defined as increase in serum creatinine (SCr) by ≥ 26.5 umol/l within 48 h, or increase in SCr to ≥ 1.5 times baseline within 7 days, or urine volume < 0.5 ml/kg/h for 6 h [24]. Skin hyperpigmentation was evaluated based on changes of the skin of the face and neck during PMB therapy or 4 weeks after treatment completion. Neurotoxicity included any of the following: apnea, encephalopathy, paresthesia, or seizures.

### Statistical analysis

Statistical analyses were carried out using the statistical software package IBM SPSS Statistics 21.0 (SPSS, Chicago, IL). Descriptive analysis was performed to describe the distribution of the variables of interest. Categorical variables were presented as counts and percentages and were compared between survivors and non-survivors using Chi-squared test or Fisher’s exact test. Continuous variables of each group were presented as the mean ± SD or median with interquartile range (IQR) and were compared between survivors and non-survivors using Student’s t-test or Mann–Whitney U test, as appropriate. Paired t-test or Wilcoxon signed rank test was used to compare the continuous variables before and after therapy and categorical variables were compared using the McNemar test. Associations between these covariates and 28-day mortality or all-cause hospital mortality were explored through multivariable logistic regression. Kaplan–Meier curves were conducted to demonstrate the survival probability within 28 days and were compared using log-rank test between groups. A *P*-value < 0.05 was considered statistically significant.

## Results

### Demographic and clinical features

A total of 106 patients were enrolled in the study between October 1, 2018 and June 30, 2019. Follow-up studies were completed for 100 patients (94.3%). The mean length of hospitalization was 41.6 ± 26.42 days (range 7–130 days), and the mean residence time in the ICU was 26 days. There were 23 patients without chronic disease and 37 patients with one chronic illness; the remainder of patients had a combination of multiple chronic diseases. There were 39 patients who had septic shock at the beginning of therapy; an additional 10 patients later progressed to shock. The demographic and clinical features of patients who received intravenous PMB are summarized in Table 1.

**Table 1:** Clinical features and details of patients receiving intravenous PMB

Characteristic	Mean ± SD, or n (%)
Age (year)	55.91 ± 17.14
Male (%)	79 (79)
ICU admission, n (%)	98 (98)
Mechanical ventilation, n (%)	49 (49)
Chronic medical conditions, n (%)	
Heart disease	14 (14)
Hypertension	44 (44)
Stroke	19 (19)
Cancer	6 (6)
Diabetes mellitus	15 (15)
Chronic obstructive pulmonary disease	1 (1)
Renal insufficiency, n (%)	27 (27)
Septic shock, n (%)	39 (39)
SOFA score, mean ± SD	7.76 ± 4.30
APACHE II, mean ± SD	17.61 ± 7.59
ICU stay before intravenous PMB, days, median (IQR)	8 (3, 14)
MODS, n (%)	60 (60)
PCT, ng/ml, median (IQR)	3.89 (1.08, 11.43)
Bacterial, n (%)	
AB	33 (33)
KP	48 (48)
PA	16 (16)
Other	9 (9)
Unknown	15 (15)
Infection sites, n (%)	
BSI	40 (40)
Pulmonary infection	64 (64)
Intraperitoneal infection	9 (9)
Incision infection	6 (6)
Others	18 (18)
Concomitant antibiotic therapy	
PMB + Carbapenem	30 (30)
PMB + Carbapenem + Tigecycline	26 (26)
PMB + Tigecycline	11 (11)
PMB + Cephalosporin	9 (9)
PMB + Carbapenem + Cephalosporin	8 (8)
Others	16 (16)
Daily dose of PMB, mg/day, n (%)	
100	53 (53)
150	17 (17)
200	30 (30)

PMB: polymyxin B; ICU: intensive care unit; SOFA: Sequential Organ Failure Assessment; APACHE II: Acute Physiology and Chronic Health Evaluation II; MODS: multiple organ dysfunction syndrome; PCT: procalcitonin; KP: *Klebsiella pneumoniae*, AB: *Acinetobacter baumannii*; PA: *Pseudomonas aeruginosa*; BSI: bloodstream infection

### Treatment regime

For 85 patients, the pathogen culture was positive; 21 patients of these were infected with two bacteria species. In 35 cases, the patient had multi-site infection. 97 patients received between 2 and 4 antimicrobials daily, and 3 patients treated with intravenous PMB as a single agent. The most common combinations were PMB + Carbapenem (30%), PMB + Carbapenem + Tigecycline (26%) and PMB + Tigecycline (11%). The other (13%) combination therapy were PMB and fosfomycin combined with carbapenem or tigecycline or cephalosporin.

Overall, the condition of patients improved after the PMB treatment. Temperature, white blood cells, C-reactive protein (CRP), procalcitonin, and APACHEII levels were significantly decreased among patients. Platelets were significantly increased (*P* < 0.001). The number of patients with mechanical ventilation or shock significantly decreased after PMB treatment (Table 2).

**Table 2:** Comparison of patient conditions before and after therapy

Parameter	Baseline	After therapy	*P*
Heart rate, bpm, mean ± SD	92.02 ± 21.55	97.36 ± 20.35	0.895
Temperature, ℃, median (IQR)	38 (37.1, 38.7)	37.1 (36.7, 37.6)	< 0.001
WBC, × 10^9^/L, median (IQR)	14.13 (10.08, 20.02)	9.28 (7.02, 13.40)	< 0.001
PLT, × 10^9^/L, mean ± SD	111.71 ± 97.68	190.95 ± 162.99	< 0.001
CRP, ug/l, median (IQR)	103.55 (56.96, 180.83)	47.6 (13.08, 102.58)	< 0.001
PCT, ng/ml, median (IQR)	3.89 (1.09, 11.43)	1.695 (0.46, 5.41)	< 0.001
SOFA, mean ± SD	7.74 ± 4.13	7.32 ± 4.41	0.282
APACHE II, mean ± SD	17.75 ± 7.69	15.98 ± 7.95	0.007
Mechanical ventilation, n (%)	51	26	< 0.001
Septic shock, n (%)	49	26	< 0.001

Categorical variables are presented as numbers (%), and continuous variables are presented as mean ± SD or interquartile ranges [IQR]

WBC: white blood cell; CRP; C-reactive protein; PLT: platelet count; PCT: procalcitonin; SOFA: Sequential Organ Failure Assessment; APACHE II: Acute Physiology and Chronic Health Evaluation II; SD: standard deviation

Microbiological eradication occurred in 66 (77.65%) out of 85 patients with electropositive germiculture. Serious adverse reactions occurred in 16 patients (16%). The rates of adverse reactions of 100, 150, 200 mg/day PMB were 15.09%, 11.76%, 20% (*P* = 0.735). Seven patients experienced at least two adverse reactions. No patients had treatment discontinued because of an adverse reaction. Nephrotoxicity was manifested by transient creatinine and urea nitrogen elevations, and no patient required hemodialysis. Among 6 patients demonstrating neurotoxicity, 4 patients showed persistent drowsiness, transient irritability, paresthesia, fatigue, dizziness, or drowsiness, and the other 2 patients underwent invasive mechanical ventilation due to adverse reactions of respiratory depression.

### Outcomes

**Fig. 1:**
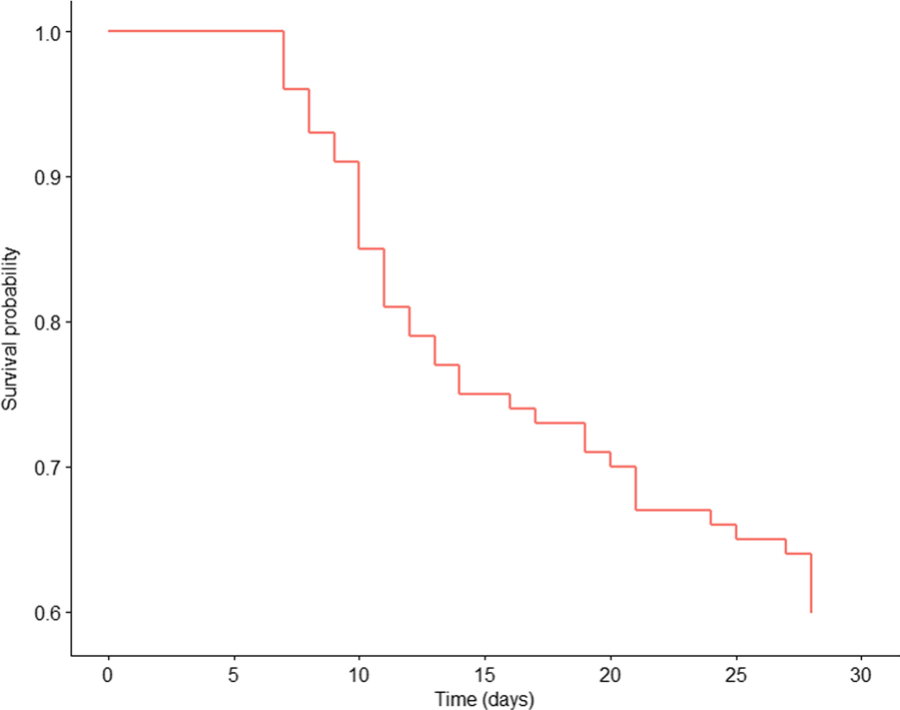
Survival analysis at 28 days: Kaplan–Meier curve. Survival rate from the receiving intravenous PMB to 28 days

The outcomes of patients receiving intravenous PMB are shown in Table 3. The 28-day mortality was 40%. More than 60% deaths occurred 7–14 days after enrollment, as shown in the Kaplan–Meier survival curve (Fig. 1). The survivors and nonsurvivors had similar characteristics (Table 4). However, the platelet count in the nonsurvivors group was lower than in the survivor group (*P* = 0.001). In terms of clinical characteristics, SOFA scores (6.77 ± 4.07 vs 9.25 ± 4.25, *P* = 0.004), APACHE II scores (16.17 ± 7.80 vs 19.7 8 ± 6.80, *P* = 0.016) and the number of patients on mechanical ventilation (21% vs 30%, *P* < 0.001) or having septic shock (17% vs 32%, *P* < 0.001) were lower in the survivor group than in the nonsurvivor group. There were no different therapeutic outcomes among the different anti-infection therapeutic regimen.

**Table 3:** Outcomes of patients receiving intravenous PMB

Outcomes	n (%)
Bacteria eradication rate, n (%)	66 (77.65)
Treatment duration, median (IQR)	11 (9, 13)
Adverse events, n (%)	16 (16)
Nephrotoxicity	7 (7)
Neurotoxicity	6 (6)
Skin hyperpigmentation	3 (3)
Eosinophil increase	7 (7)
ICU mortality, n (%)	12 (12)
Hospital mortality, n (%)	15 (15)
28-day mortality, n (%)	40 (40)

**Table 4:** Characteristics associated with 28-day mortality among patients who received intravenous PMB

Parameter	Value for:		*P*
	Survivors (n = 60)	Nonsurvivors (n = 40)	
Age, y, mean ± SD	54.65 ± 16.23	57.8 ± 18.45	0.370
Female, n (%)	47 (78.3)	32 (80.0)	0.840
WBC, × 10^9^/L, median (IQR)	14.28 (10.58, 20.44)	13.84 (9.55, 19.65)	0.359
PLT, × 10^9^/L, mean ± SD	131.37 ± 101.96	71.15 ± 66.27	0.001
PCT, ng/ml, median (IQR)	2.30 (0.70, 10.07)	5.00 (1.97, 15.93)	0.062
CRP, ug/l, median (IQR)	100.15 (50.70, 174.33)	119.96 (79.58, 186.0)	0.624
SOFA, mean ± SD	6.77 ± 4.07	9.25 ± 4.25	0.004
APACHE II, mean ± SD	16.17 ± 7.80	19.78 ± 6.80	0.016
Mechanical ventilation, n (%)	21 (35.0)	30 (75.0)	< 0.001
Septic shock, n (%)	17 (28.3)	32 (80.0)	< 0.001
Hospitalization before intravenous PMB, days, median (IQR)	12.00 (5.25, 20.75)	11.50 (3.25, 21.00)	0.882
ICU stay before intravenous PMB, days, median (IQR)	9.00 (3.00, 15.00)	5.00 (1.00, 14.00)	0.156
Treatment duration, days, median (IQR)	11.00 (9.00, 13.00)	10.00 (8.25, 13.75)	0.697
Concomitant antibiotic therapy, n (%)			0.844
PMB + Carbapenem	19 (31.67)	11 (27.5)	
PMB + Carbapenem + Tigecycline	16 (26.67)	10 (25)	
PMB + Tigecycline	6 (10)	5 (12.5)	
PMB + Cephalosporin	4 (6.67)	5 (12.5)	
PMB + Carbapenem + Cephalosporin	4 (6.67)	4 (10)	
Others	11 (18.33)	5 (12.5)	
Daily dose of PMB, mg/day, n (%)			0.425
100	35 (58.33)	18 (45)	
150	9 (15)	8 (20)	
200	16 (26.67)	14 (35)	
Identify microorganisms, n (%)	52 (86.67)	33 (82.5)	0.580
KP, n (%)	32 (53.3)	16 (40.0)	0.191
AB, n (%)	19 (31.7)	14 (35)	0.728
Multisite infection, n (%)	20 (33. 3)	15 (37.5)	0.669
Pulmonary infection, n (%)	35 (58.3)	29 (72.5)	0.217
BSI, n (%)	26 (43.3)	14 (35.0)	0.405
Chronic medical conditions, n (%)	45 (75.0)	32 (80.0)	0.734
Nephrotoxicity, n (%)	13 (21.7)	13 (32.5)	0.251
Adverse reactions, n (%)	8 (13.3)	8 (20.0)	0.373

Categorical variables are presented as numbers (%), and continuous variables are presented as mean ± SD or interquartile ranges [IQR]

WBC: white blood cell; PLT: platelet count; PCT: procalcitonin; CRP: C-reactive protein; SOFA: Sequential Organ Failure Assessment; APACHEII: Acute Physiology and Chronic Health Evaluation II; PMB: polymyxin B; ICU: intensive care unit; KP: *Klebsiella pneumoniae*; AB: *Acinetobacter baumannii*; BSI: Bloodstream Infection; SD: standard deviation

Prior to intravenous PMB, empirically anti-infective treatment by intravenous carbapenem or tigecycline 10.4 ± 8.3 days (range 2–28 days) showed no clinical improvement. Efforts to improve salvage treatment were urgently required. The mortality among the 85 patients with identified pathogens was 38.82%, while the mortality among patients with negative pathogen culture results was 46.67% (*P* = 0.58). During treatment, similar adverse reactions related to PMB were observed in the two groups. There were no significant differences in characteristic and therapeutic effect of patients with intravenous PMB among centers (Table 5).

**Table 5:** Characteristic and therapeutic effect of patients receiving intravenous PMB among centers

	Value for:		*P*
	Sub-centers (n = 83)	Main center (n = 17)	
Mechanical ventilation, n (%)	44 (53.01)	7 (41.18)	0.432
Septic shock, n (%)	40 (48.19)	9 (52.94)	0.721
Adverse events, n (%)	14 (16.87)	2 (11.76)	0.601
Non-survivors, n (%)	33 (39.75)	7 (41.18)	0.222

**Fig. 2:**
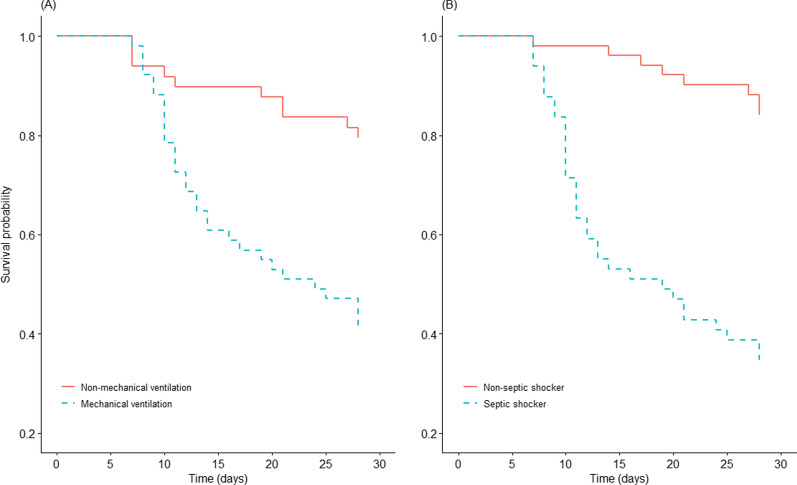
Kaplan–Meier 28-day survival curve comparing patients on mechanical ventilation or with septic shock. **A** 28-day mortality rate was 59% and 20% in patients with mechanical ventilation and not on mechanical ventilation, respectively (log-rank, *P* < 0.01). **B** 28-day mortality rate was 65% and 16% in patients with septic shock and without septic shock, respectively (log-rank, *P* < 0.01)

The factors demonstrating statistically significant differences in univariate analysis in Table 4 were analyzed by logistic regression (Table 6). The 28-day mortality was 58.82% for patients on mechanical ventilation compared to 20.41% for patients who were not on mechanical ventilation. The 28-day mortality was 65.31% in patients with septic shock compared to 15.69% in patients who did not have septic shock. Survival of patients is shown in Fig. 2.

**Table 6:** Multivariate analysis of factors associated with 28-day mortality

Variable	OR (95% CI)	*P*-value
PLT	1.578 (0.965–2.580)	0.069
Mechanical ventilation	3.580 (1.194–10.739)	0.023
Septic shock	5.960 (1.923–18.473)	0.002
APACHE II	1.013 (0.613–1.673)	0.960
SOFA	0.941 (0.553–1.601)	0.823

OR: odds ratio; CI: confidence interval; PLT: platelet count; APACHE II: Acute and Physiology and Chronic Health Evaluation; SOFA: Sequential Organ Failure Assessment

## Discussion

In this study, patients with CR-GNB infection treated with PMB had a bacteria eradication rate of 77.65%, ICU-related mortality of 12%, hospital mortality of 15%, and 28-day mortality of 40%. These results are favorable compared with those reported for studies of patients receiving different treatments for CR-GNB infections [25]. For example, in patients infected with carbapenem-resistant *Acinetobacter baumannii* who did not receive appropriate empirical antimicrobial therapy, the overall mortality rate was 86.1% [26]. In one retrospective study, the overall ICU mortality rate was 45.2% for critically ill patients infected with CR-GNB who received tigecycline therapy [27]. In another retrospective study, the in-hospital mortality rate for patients receiving tigecycline for the treatment of carbapenem-resistant *Klebsiella pneumoniae* bloodstream infections was 62.5% [28]. In a retrospective cohort study of the treatment of infections due to carbapenem-resistant Enterobacteriaceae, the 30-day mortality was 50% after ceftazidime–avibactam treatment [29]. Our results suggest that treatment with PMB may reduce the mortality of patients with CR-GNB.

Our study found that after 7 days of PMB treatment, the temperature of patients with CR-GNB infection returned to normal, and the number of patients with septic shock or mechanical ventilation decreased. Moreover, the infection indicators of white blood cell, procalcitonin, and CRP were all significantly reduced. The symptoms of thrombocytopenia in patients with CR-GNB infection improved. APACHE II scores were also lower than the initial sepsis. The mortality of PMB target therapy was 38.82%, while the mortality of patients with empiric therapy was 46.67% (*P* = 0.580). Our results suggest that PMB may be a clinically effective treatment for patients infected with CR-GNB. The survival was no difference in 28-day mortality in patients that received 100, 150, 200 mg/day PMB. Some studies found that there was no difference in PMB daily dose between survivors and nonsurvivors [20, 30]. But other studies suggested that the daily dose of PMB treatment failure in CR-GNB infections among critically ill patients was lower than success [9]. Randomized controlled trials with large data are required to determine the optimal therapeutic dose.

Our results suggest that PMB therapy is safe for the treatment of infections caused by CR-GNB, as intravenous PMB was well tolerated in most patients. Serious adverse reactions occurred in 16 patients (16%), 7 patients had at least two adverse reactions, eight of whom had transient adverse reactions.

In our study, adverse effects related to nephrotoxicity occurred in 7 patients (7%) and were mild and reversible; none required renal replacement therapy. While the prevalence of nephrotoxicity was not lower in our study than in other observational studies (4.2% to 40.5%) [11, 18, 20, 31–34], it was at the lower end of these reported ranges. Assessment of the contribution of PMB to renal impairment may be complicated by other factors such as infection, septic shock, multiple organ dysfunction syndrome (MODS), and concomitant use of other nephrotoxic drugs. One study found that malignancy, co-infection with other microorganisms, and PMB daily dose were risk factors for polymyxin B-associated nephrotoxicity [35]. Their results also indicated daily dose differed between patients with and without nephrotoxicity (131 mg vs 150 mg, *P* = 0.005) during PMB administration [35]. Our results showed that PMB daily dose was not marked relation with adverse effects.

Neurotoxicity of PMB is less common than nephrotoxicity, and it is usually mild and resolves after prompt discontinuation of therapy [11, 20, 31]. In another study, however, no cases of neurotoxicity complications occurred among 247 patients who were given PMB therapy [31]. In a previous study, intravenous PMB was associated with neurotoxicity (7%), manifestations of seizures and neuromuscular weakness [11]. In our study, among the 6 patients with neurotoxicity, 2 patients underwent invasive mechanical ventilation due to the adverse effects of respiratory depression. Neither patient used sedative analgesics. PMB therapy was not stopped in the patients with respiratory depression and the endotracheal tube was removed before the end of PMB treatment.

In the present study, three cases (3%) developed skin hyperpigmentation in the face and neck. This incidence is lower than previous studies [20, 31]. During treatment, there were no other adverse reactions such as rashes, itching, dermatitis, or fever. While our results support the use of PMB, the safety of polymyxin therapy requires further study.

Mechanical ventilation and septic shock were associated with higher 28-day mortality in the present study. Similarly, the other study showed mechanical ventilation, septic shock, multiple-site infection, and total PMB cumulative dose to be independently associated with treatment efficacy [20]. A previous study of a large US cohort found that most patients with culture-positive community onset sepsis did not have resistant bacteria [36], while a study of the epidemiology of sepsis in Chinese ICUs found that only 12% of culture-positive were multi-drug resistant organisms [37]. In our study, the mortality of patients with empiric PMB therapy was higher. This underscores the need for rapid identification of CR-GNB infection and an increased of judicious use of PMB for the treatment of sepsis, to avoid progression to mechanical ventilation or septic shock. In this regard, the optimization and validation of PMB-based combinations may have considerable clinical benefits. Our combination therapy was similar to that of most other research [9, 20]. It has been reported that the development of resistance was observed over a course of 72 h with PMB monotherapy against CRAB isolates [38]. Several clinical trials demonstrated that the increased use of colistin has led to the development of colistin resistance. It is suggested that intravenous of colistin, carbapenem, quinolone in the past three months prior to hospitalization and the length of hospital stay were risk factors contributing to colistin-resistant against microorganisms infection [39].Therefore, rational use of the colistins will be essential.

The present study has several limitations, including its retrospective, real-world design and lack of a control group or direct basis of comparison with other treatments. Another limitation was the relatively small number of patients included in the study. Serum PMB concentrations were not determined in the study. The decision to use additional antibiotics was made by individual clinicians, which may have introduced bias. Additionally, the concomitant use of other antibiotics with PMB makes it impossible to attribute treatment efficacy solely to PMB. Further, it was difficult to properly evaluate adverse effects attributable to PMB in view of the use of other drugs in seriously ill patients. Despite these limitations, our study represents a multicenter study evaluating a range of CR-GNB infections treated with PMB combination therapy.

## Conclusions

In summary, the findings from our study suggest that timely and appropriate use of PMB may have a positive impact on clinical outcomes in the treatment of CR-GNB infections. These results underscore the need to more quickly identify patients with CR-GNB who may benefit from judicious use of PMB, in particular, patients with septic shock or on mechanical ventilation who may be at higher risk of mortality. Clinicians should apply strict protocols when using this antimicrobial agent to prevent the occurrence and spread of polymyxin resistance.

## Data Availability

The datasets used and/or analyzed in this study are not publicly available due to privacy issues but are available from the corresponding author upon reasonable request.

## Abbreviations

CR-GNB: Carbapenem-resistant Gram-negative bacilli
PMB: Polymyxin B
ICU: Intensive care unit
APACHEII: Acute Physiology and Chronic Health Evaluation II
SOFA: Sequential Organ Failure Assessment
HAP: Hospital-associated pneumonia
VAP: Ventilator-associated pneumonia
PCT: Procalcitonin
CRP: C-reactive protein
PLT: Platelet count
MODS: Multiple organ dysfunction syndrome
